# Hold-and-drag suturing using a new closure device

**DOI:** 10.1055/a-2335-6761

**Published:** 2024-06-25

**Authors:** Hiroki Kato, Makoto Kobayashi, Hitoshi Sugiyama

**Affiliations:** 1 Department of Gastroenterology, Yokkaichi Municipal Hospital, Yokkaichi, Japan

Various suturing techniques have been used to close mucosal defects after endoscopic resection 1 2 3 . Hold-and-drag suturing, in which one side of the mucosa is grasped with a graspable endoscopic clip and pulled to the contralateral mucosa for suturing, is highly convenient because it does not require other assistive devices. However, if the tip of the clip does not catch well, it will come off when the hook is opened.

The newly introduced Mantis closure device (hereafter “Mantis”; Boston Scientific, Marlborough, Massachusetts, USA) ( Fig. 1 , Fig. 2 ) allows for rotation and reattachment, and the jaws at the tip are sharp and point slightly inward to ensure a secure hold-and-drag maneuver. This study used the Mantis closure device to examine the hold-and-drag suture technique ( Video 1 ).

**Fig. 1:**
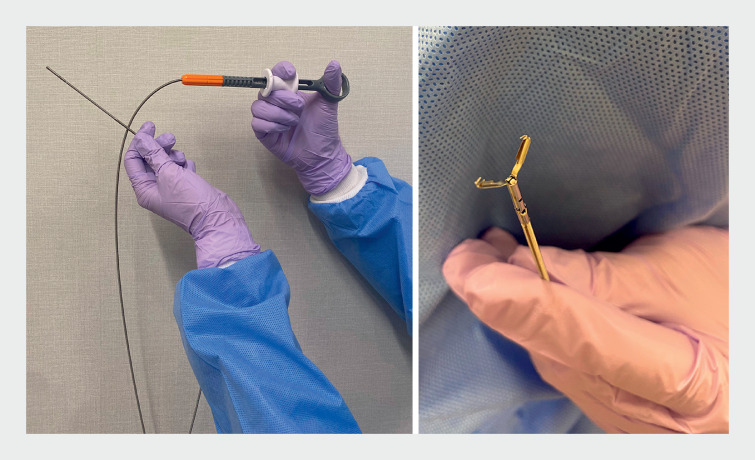
The Mantis closure device (Boston Scientific, Marlborough, Massachusetts, USA).

**Fig. 2:**
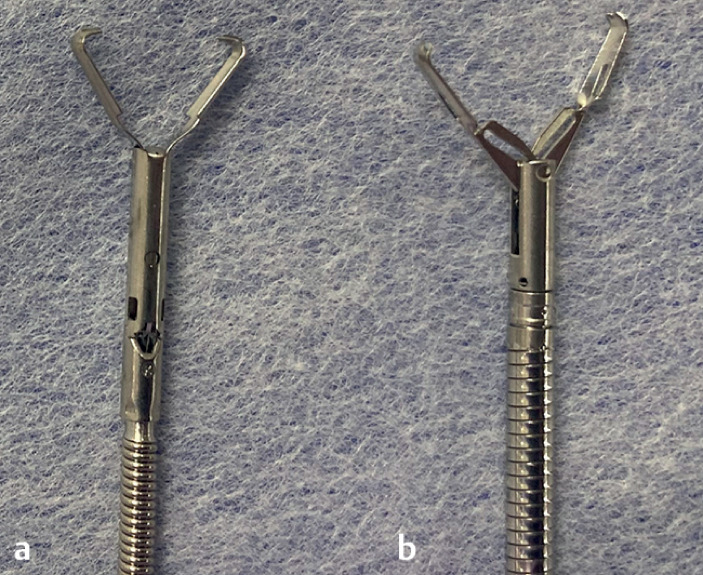
Comparison of clips. **a** The Mantis closure device (Boston Scientific, Marlborough, Massachusetts, USA). **b** The SureClip (16 mm; Micro-Tech Co. Ltd, Nanjing, China).

Hold-and-drag suturing using a new closure device (Mantis closure device; Boston Scientific, Marlborough, Massachusetts, USA). This clip is excellent for gripping mucous membranes.Video 1

Four patients undergoing endoscopic submucosal dissection (ESD) of the cecum, transverse, sigmoid colon, and rectum, respectively, were included in the study.

First, the center of one side of the mucous membrane was grasped with the Mantis and closed. The tip was then shaken slightly to allow the claw to bite into the mucous membrane fully. Next, the inside of the lumen was sufficiently aspirated. Then, the contralateral mucous membrane stump was brought close, and the Mantis clip was opened and grasped the opposite membrane before being closed. The mucous membrane was sutured at both ends and then the entire defect was completely closed using standard clips.

The median lesion resection diameter was 21.5 mm (range 30–15 mm). The median time for the hold-and-drag suture was 51 seconds (range 61–44 seconds) and the median overall suture time was 364 seconds (range 510–201 seconds). No complications, such as postoperative bleeding or perforation, were observed.

The sharp, inward-facing claws enabled secure grasping of the mucosa, and there was no mucosal loss during opening or damage to the mucosa during hold-and-drag owing to the chamfer in the center of the claws.

The Mantis is considered very useful for hold-and-drag suturing after colorectal ESD.

Endoscopy_UCTN_Code_TTT_1AQ_2AK

## Notes

Endoscopy E-Videos https://eref.thieme.de/e-videos
*E-Videos* is an open access online section of the journal *Endoscopy* , reporting on interesting cases and new techniques in gastroenterological endoscopy. All papers include a high-quality video and are published with a Creative Commons CC-BY license. Endoscopy E-Videos qualify for HINARI discounts and waivers and eligibility is automatically checked during the submission process. We grant 100% waivers to articles whose corresponding authors are based in Group A countries and 50% waivers to those who are based in Group B countries as classified by Research4Life (see: https://www.research4life.org/access/eligibility/ ). This section has its own submission website at https://mc.manuscriptcentral.com/e-videos .
